# ChatGPT as a Learning Tool for Medical Students: Results From a Randomized Controlled Trial

**DOI:** 10.7759/cureus.85767

**Published:** 2025-06-11

**Authors:** Kazi A Kalam, Fadi D Masoud, Adam Muntaser, Raghav Ranga, Xue Geng, Munish Goyal

**Affiliations:** 1 Department of Medicine, Georgetown University School of Medicine, Washington, DC, USA; 2 Department of Emergency Medicine, Wayne State University, Detroit, USA; 3 Department of Biostatistics, Bioinformatics and Biomathematics, Georgetown University, Washington, DC, USA; 4 Department of Emergency Medicine, MedStar Washington Hospital Center, Washington, DC, USA

**Keywords:** artifical intelligence, chatgpt, generative ai, medical school education, randomized controlled trial

## Abstract

Importance: As artificial intelligence (AI) tools like ChatGPT become increasingly widespread in medical education, it is essential to evaluate the effectiveness of AI tools in enhancing students' academic performance and retention compared to traditional educational resources such as lecture materials and textbooks.

Objective: We aim to determine whether ChatGPT-4.0 improves medical students' short-term academic performance compared to internal institutional resources and publicly available online materials.

Design: This study was a single-center, prospective, randomized controlled trial conducted over a two-week period in April 2025.

Setting: The research took place at Georgetown University School of Medicine.

Participants: A total of 198 first-year medical students were invited to participate, with 33 students enrolling in the study. Participants (N = 33) were assigned to one of three groups: Group A (ChatGPT-4.0), Group B (external resources, including publicly available online materials such as Google, PubMed, and third-party educational websites, but excluding AI-assisted tools), and Group C (institutional resources such as electronic textbooks and lecture materials).

Interventions: Individuals (N = 33) were randomly assigned to one of three groups: Group A (ChatGPT-4.0; N = 10, 30.3%), Group B (external resources; N = 12, 36.4%), and Group C (institutional resources; N = 11, 33.3%). Participants completed an initial multiple-choice quiz using their assigned resources, followed by a post-quiz survey. One week later, they retook the same quiz without access to any resources to assess retention.

Primary outcome(s) and measure(s): The primary outcome was the initial quiz score. The secondary outcome was the retention score, evaluated through the second quiz without resources.

Results: Initial quiz scores (Week 1) were significantly higher in Group A (N = 10, mean = 9.60 ± 0.52) and Group B (N = 12, mean = 9.08 ± 0.79) compared to Group C (N = 11, mean = 6.64 ± 1.57) (p < 0.001). However, retention scores one week later (Week 2) showed no significant differences among the groups: Group A (N = 10, mean = 6.20 ± 1.93), Group B (N = 12, mean = 5.58 ± 2.07), and Group C (N = 11, mean = 4.36 ± 2.01) (p = 0.118).

Conclusions and relevance: ChatGPT-4.0 improves short-term academic performance but does not provide a short-term retention advantage over institutional or external online educational resources. These findings demonstrate the potential of AI tools to enhance short-term learning outcomes while emphasizing the need for further research to evaluate their long-term effectiveness in educational settings.

## Introduction

General context of artificial intelligence (AI) in medical education

Artificial intelligence (AI) is transforming education by providing tools that replicate human reasoning, learning, and decision-making [1]. Among the most impactful innovations is generative AI, which creates new content through text, images, or code in response to prompts [2]. ChatGPT, a generative AI model developed by OpenAI, exemplifies this technology. It employs transformer-based machine learning to generate real-time, context-aware responses [3,4]. In medical education, where students face intense cognitive demands, ChatGPT has emerged as a tool that offers personalized, on-demand explanations [5]. These features suggest that it could augment traditional learning methods by improving access to information and enhancing engagement. As interest in the role of AI in education continues to grow, it is essential to evaluate how tools like ChatGPT influence medical student performance and knowledge retention [6].

Cognitive Load Theory (CLT) as a theoretical framework

Cognitive Load Theory (CLT) posits that learning is enhanced when working memory demands are effectively managed [7]. CLT categorizes cognitive load into three types: intrinsic (the inherent complexity of the material), extraneous (how the material is presented), and germane (the mental effort needed to build meaningful knowledge structures) [7,8].

In medical education, intrinsic load is inherently high due to the complexity and density of the content. Poorly organized materials, such as dense textbooks or unclear lectures, can elevate extraneous load and impede learning [8]. Tools like ChatGPT may help alleviate this by providing immediate, clear explanations that facilitate access to key concepts [9].

Traditionally, medical education relies on lectures, textbooks, and external resources. Although these methods can be effective, they may not always meet individual learning needs. In contrast, ChatGPT offers real-time, personalized responses that adapt to a student's pace. Reducing extraneous load may free cognitive resources for relevant processing, potentially improving understanding and retention [10].

Existing literature and broader discussion of AI in medical education and research gap

Recent studies have explored AI's role in medical education. One study described ChatGPT as an interactive "study buddy," providing real-time feedback and generating clinical scenarios, which may enhance access for students with limited educational resources [2]. Another found that ChatGPT performed at or near the passing threshold on the United States Medical Licensing Examination (USMLE), suggesting its potential for on-demand learning [11].

A randomized controlled trial conducted in Chinese orthopedic education demonstrated that the use of ChatGPT led to improved short-term performance and examination scores when compared to traditional learning tools [12]. However, that study lacked a controlled setting and concentrated on a single specialty. Overall, there are few empirical studies that have rigorously tested AI's effectiveness across disciplines or assessed its impact on retention [13-15].

Our study addresses this gap by evaluating the impact of ChatGPT-4.0 on short-term performance and retention across various subjects. Furthermore, we compare it with external resources (e.g., Google and PubMed) and internal institutional resources (e.g., lectures and electronic textbooks) while capturing student perceptions of AI's educational value.

Study objectives and hypothesis

This study evaluates whether ChatGPT-4.0 enhances medical students' short-term academic performance and knowledge retention over a brief interval compared to institutional educational resources. We hypothesize that ChatGPT-4.0 will improve immediate performance by reducing extraneous cognitive load, enabling students to focus more effectively on core learning objectives. However, we also explore whether these short-term benefits extend to retention after one week, acknowledging that generative AI tools may limit the depth of cognitive processing necessary for long-term knowledge consolidation [10], which was not tested in our study.

The study objectives are as follows: (1) evaluate short‑term academic performance by comparing initial quiz scores among students using ChatGPT, external online resources, and internal institutional resources; (2) assess short‑term knowledge retention by comparing Week 2 quiz scores, taken without resources, among the same three groups; (3) interpret any performance and retention differences within the framework of Cognitive Load Theory; and (4) collect and analyze student perceptions of each resource's usefulness, efficiency, and accuracy through structured post‑quiz surveys.

## Materials and methods

Study design

This single-center, prospective randomized controlled trial was conducted at Georgetown University School of Medicine to compare medical student performance and retention using three types of resources: ChatGPT-4.0, external sources (e.g., Google and PubMed), and internal institutional materials (e.g., lectures and electronic textbooks). The two-week study included an initial quiz, followed by a repeat of the same quiz one week later to assess retention.

Participants and recruitment

In April 2025, first-year Doctor of Medicine (MD) students at Georgetown University School of Medicine who were in good academic standing were recruited via email and campus bulletin board flyers. Inclusion criteria were enrollment in the first-year MD curriculum, good academic standing as verified by the Office of Student Affairs, and provision of written informed consent; no exclusion criteria were applied. Participation was incentivized with a weekly drawing for a $100 gift card. Institutional review board (IRB) approval and consent procedures are described in the Ethical Considerations section.

Interventions

Participants were randomly assigned to one of three groups: Group A had access to ChatGPT-4.0; Group B had access to external resources (these include publicly available online materials, e.g., Google, PubMed, and third-party educational websites), excluding AI-assisted tools; and Group C had access to internal institutional resources, such as lecture materials, electronic textbooks, and course-provided slides.

Quizzes were delivered electronically via Qualtrics XM (Qualtrics, Provo, UT), and all 10 multiple-choice items were adapted from faculty-approved formative assessments aligned with course learning objectives. Participants in Group A accessed ChatGPT-4.0 (OpenAI GPT-4.0 model, April 2025 release) through the standard web interface. ChatGPT was available for ad hoc queries during the quiz but was not configured to deliver structured retrieval practice or spaced repetition prompts. All groups completed the same quiz questions under identical timing conditions. In Week 1, each group completed an identical 15-minute proctored quiz using only their assigned resources. In Week 2, the same quiz was readministered without any resource access to assess short-term knowledge retention.

Randomization and blinding

Randomization was performed using a pseudo-random number generator. Participants and researchers were not blinded to group assignments.

Outcome measures

The primary outcome was the initial quiz score, which assessed students' performance based on their use of assigned study resources during the assessment. The quiz consisted of 10 multiple-choice questions covering key concepts in pathology, pharmacology, physiology, and anatomy, drawn from the first-year medical curriculum. These topics reflected material that students were expected to have mastered at this point in the semester. All questions were aligned with course learning objectives and adapted from formative assessments developed by course instructors to evaluate student understanding, thereby supporting content validity. 

Secondary outcomes included knowledge retention, measured by the score on a repeat quiz administered one week later using the same set of questions. Quiz completion time and student perceptions were also collected through surveys administered after each quiz. These surveys captured subjective feedback on the perceived usefulness, effectiveness, and overall experience of using the assigned resources.

Survey instruments

Participants from each group completed structured surveys after both quiz encounters. These surveys were developed specifically for this study and included items designed to assess student perceptions. Following the initial quiz, participants evaluated the assigned resources in terms of usefulness, satisfaction, effectiveness, understanding, confidence in accuracy, comfort, information quality, and perceived efficiency. After the second quiz, the survey focused on perceived retention, quiz difficulty, and the resource's impact on retention.

All surveys were administered electronically via Qualtrics using QR codes and included Likert-scale items assessing the domains described above. Survey results by group are presented in the Appendices.

Statistical analysis

Descriptive analyses were performed using frequencies and percentages for categorical variables and means ± standard deviations (SD) for continuous variables. All analyses were performed in RStudio (version 2024.04.1) (Posit Software, Boston, MA). Assumptions of normality and homogeneity of variance were evaluated with the Shapiro-Wilk test and Levene's test (car package), respectively.

A one‑way analysis of variance (ANOVA) compared continuous outcomes across the three study groups, and Tukey's honestly significant difference (HSD) test provided pairwise comparisons for initial quiz scores (Week 1). For categorical variables, differences were assessed with Fisher's exact test, followed by pairwise Fisher's exact tests when overall results were significant. To control the false‑discovery rate for multiple comparisons, the Benjamini-Hochberg procedure was applied.

Effect sizes for ANOVA are reported as eta‑squared (η²), and proportions are provided for categorical results. All statistical tests were two‑sided, with p < 0.05 considered statistically significant.

Ethical considerations

The study protocol was reviewed by the Georgetown University Institutional Review Board and determined to be exempt under Category 2(ii), covering research involving educational tests and surveys. Informed consent was obtained from all participants. Each participant was assigned a numeric study ID; identifiers were stored separately from quiz and survey data, which resided on an encrypted Qualtrics server protected by two-factor authentication. De-identified CSV files were exported to a password-protected institutional drive accessible only to the study statistician and the corresponding author. Quiz results were additionally secured behind Duo Access authentication to ensure confidentiality.

## Results

**Figure 1:**
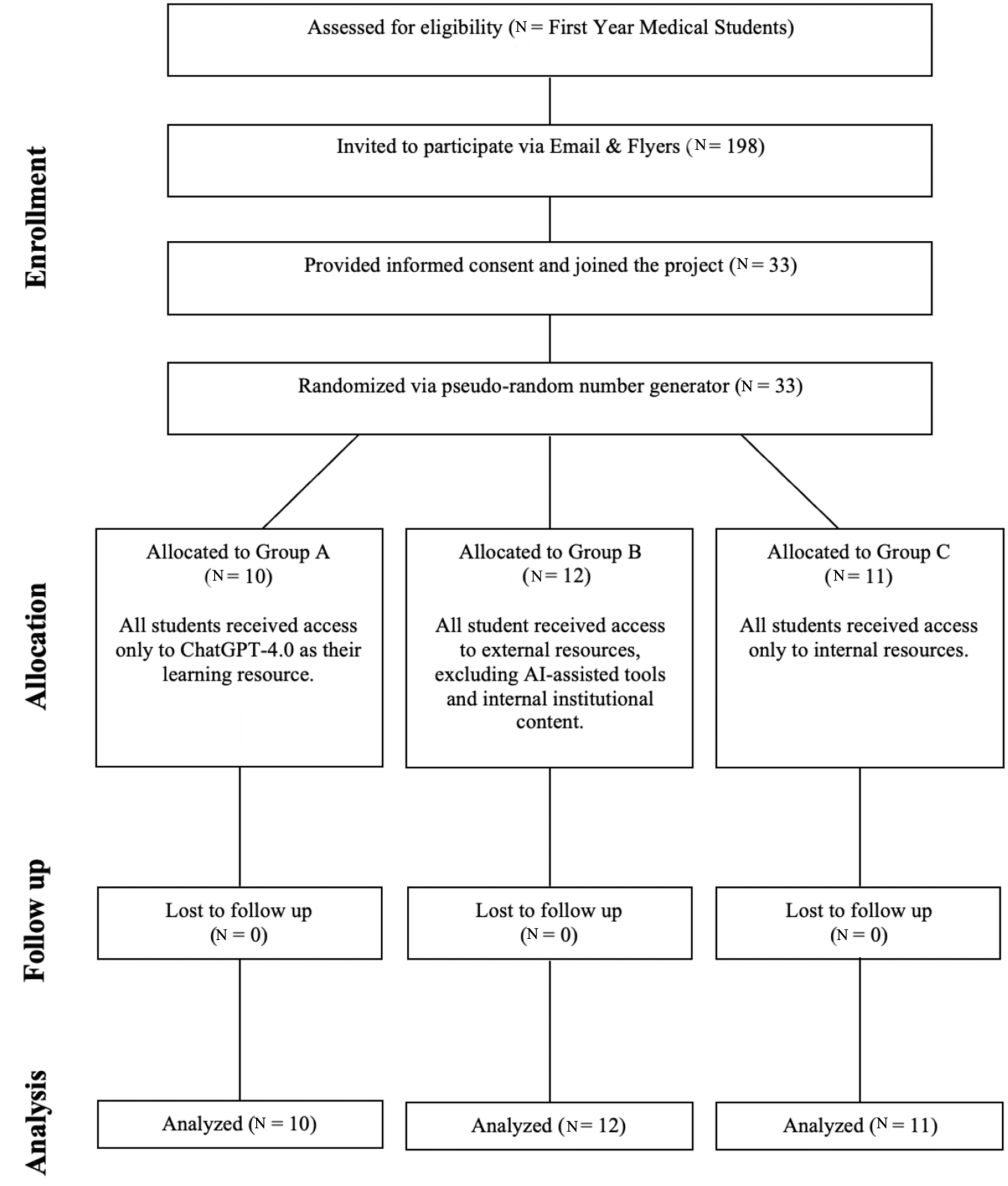
CONSORT Flow Diagram Flow diagram illustrating participant enrollment, randomization into three groups, quiz completion, and follow-up. Data are presented as N. No statistical tests were applied. This figure summarizes participant flow through each phase of the study. CONSORT: Consolidated Standards of Reporting Trials

A total of 33 students were distributed across three groups: Group A (ChatGPT-4.0; N = 10, 30.3%), Group B (external resources; N = 12, 36.4%), and Group C (institutional resources; N = 11, 33.3%) (Table 1). Figure 1 illustrates the flow of participants through each stage of the study, including enrollment, group allocation, quiz completion, and follow-up.

**Table 1:** Group Allocation Summary: Distribution of 33 Students Across Groups A, B, and C Data are presented as numbers (%), where percentages indicate within-group proportions. No statistical tests were applied.

Category	Overall (N = 33)	Group A (N = 10)	Group B (N = 12)	Group C (N = 11)
Number of students	33	10	12	11
Group allocation (%)				
A	10 (30.3)	10 (100.0)	0 (0.0)	0 (0.0)
B	12 (36.4)	0 (0.0)	12 (100.0)	0 (0.0)
C	11 (33.3)	0 (0.0)	0 (0.0)	11 (100.0)

**Figure 2:**
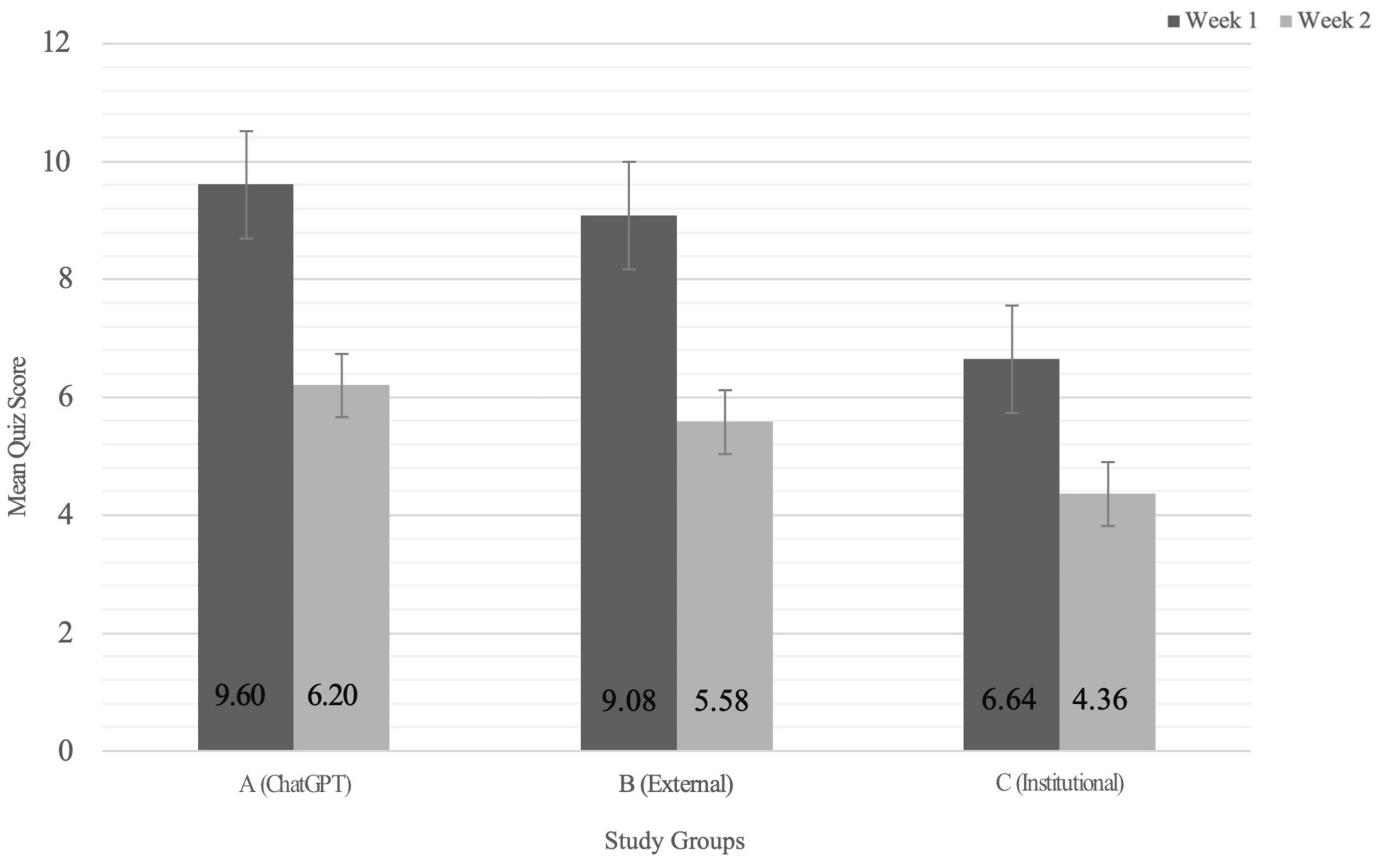
Average Quiz Scores by Group During Week 1 (With Resources) and Week 2 (Without Resources) Data are presented as means. Error bars represent SD. Sample sizes were N = 10 (Group A), 12 (Group B), and 11 (Group C). P-values were calculated using one-way ANOVA, with statistical significance defined as p < 0.05. SD: standard deviation, ANOVA: analysis of variance

Initial mean quiz scores differed significantly among the groups: Group A (N = 10, mean = 9.60 ± 0.52), Group B (N = 12, mean = 9.08 ± 0.79), and Group C (N = 11, mean = 6.64 ± 1.57) (p < 0.001). These performance trends across Week 1 and Week 2 are illustrated in Figure 2. Post hoc analysis revealed that Group A and Group B scored significantly higher than Group C (p < 0.001), while the difference between Group A and Group B was not statistically significant (p = 0.50).

Delayed mean repeat quiz scores revealed a nonsignificant trend toward improved retention: Group A (N = 10, mean = 6.20 ± 1.93), Group B (N = 12, mean = 5.58 ± 2.07), and Group C (N = 11, mean = 4.36 ± 2.01) (p = 0.118) (Table 2 and Figure 2). Regarding quiz completion times, Group A completed the Week 1 quiz in an average of 8.92 minutes, Group B in 8.83 minutes, and Group C in 10.04 minutes. There was no significant difference in completion times during Week 1 (p = 0.505) (Table 2). In Week 2, Group A averaged 2.34 minutes (SD = 1.07), Group B 2.38 minutes (SD = 1.62), and Group C 2.03 minutes, with no significant differences across groups (p = 0.771) (Table 2), indicating no measurable difference in efficiency.

**Table 2:** Group Quiz Performance and Completion Time During Week 1 (With Resources) and Week 2 (Without Resources) Quiz scores are reported as mean ± SD. Quiz completion times are reported in seconds. f-values and p-values were calculated using one-way ANOVA. Statistical significance was defined as p < 0.05. SD: standard deviation, ANOVA: analysis of variance

Variable	Group A (ChatGPT)	Group B (External)	Group C (Institutional)	f-value	p-value
Quiz score (Week 1)	9.60 ± 0.52	9.08 ± 0.79	6.64 ± 1.57	f(2,30) = 24.01	p < 0.001
Quiz completion time (Week 1)	534.90 ± 168.60	539.00 ± 179.10	602.60 ± 132.10	f(2,30) = 0.70	p = 0.505
Quiz score (Week 2)	6.20 ± 1.93	5.58 ± 2.07	4.36 ± 2.01	f(2,30) = 2.3011	p = 0.118
Quiz completion time (Week 2)	140.00 ± 64.00	142.92 ± 97.22	121.55 ± 56.41	f(2,30) = 0.262	p = 0.771

Similarly, there was no significant difference among groups in their comfort using the assigned resources as a primary method of medical education: Group A (N = 10), Group B (N = 12), and Group C (N = 11) (p = 0.368). Perceived retention of medical concepts after the first encounter also did not differ significantly among groups (p = 0.340).

Post hoc power analysis

A post hoc power analysis was conducted for each outcome. The initial quiz scores (Score 1) (Table 2) comparison showed 99% power at α = 0.05, indicating sufficient power to detect differences. Based on the observed effect size (f* *= 1.24), a future study would require only 12 participants to achieve 80% power. In contrast, the analysis of retention scores (Score 2) exhibited 43% power (f = 0.37), with an estimated required sample size of 72 for adequate power. The difference between Score 2 and Score 1 revealed only 25% power (f* *= 0.276), necessitating 132 participants to reach 80% power. These findings highlight that while our study was adequately powered for the primary outcome, it may have been underpowered for retention-related outcomes.

## Discussion

Our findings closely align with our initial hypothesis. Students using ChatGPT-4.0 scored significantly higher on initial quizzes compared to those utilizing institutional resources, supporting our prediction that ChatGPT may reduce extraneous cognitive load, enabling learners to grasp immediate learning objectives more efficiently [8]. There was no statistically significant difference between the ChatGPT group and the external resources group, suggesting a comparable advantage in learning outcomes between ChatGPT and external materials.

In terms of retention, our hypothesis predicted uncertainty, theorizing that generative AI could inadvertently decrease germane cognitive load, potentially hindering the deeper cognitive engagement essential for sustained retention [7]. Consistent with this prediction, we observed no significant differences in retention across groups. While students initially benefited from ChatGPT's streamlined information delivery, the generative AI approach may have diminished the active cognitive processing (germane load) necessary for robust knowledge consolidation [9,16]. ChatGPT's use for immediate information lookup may have failed to engage the retrieval and elaborative processes known to bolster retention, which could account for the absence of a statistically significant retention effect. It is worth noting that while the initial quiz scores were sufficiently powered to detect significant differences between groups, the comparisons related to knowledge retention (Score 2) and change in performance over time (Score 2-Score 1), as shown in Table 2, were underpowered. This may partially explain the lack of statistically significant differences in long-term retention outcomes despite numerical trends favoring the ChatGPT group. Factors such as a short study duration and limited sample size might have influenced these findings, indicating the need for future studies employing longer follow-up periods and larger cohorts to clarify generative AI's impact on long-term retention [10].

This finding aligns with prior research, demonstrating enhanced short-term performance when utilizing AI tools in particular medical fields [12,13]. The significant differences observed in post hoc analyses (Group A: N = 10, Group B: N = 12, Group C: N = 11) indicate that both ChatGPT and external resources provided a stronger foundation for short-term academic performance during the initial quiz (Week 1) compared to institutional resources such as lectures, slides, and textbooks. Traditional lecture slides and textbooks, when used in isolation, appeared to offer less immediate cognitive support than AI-based or carefully curated external resources, emphasizing the need to modernize institutional materials or pair them with interactive learning aids.

These advantages did not extend to short-term retention. While Group A (N = 10) scored higher than Group B (N = 12) and Group C (N = 11) on the follow-up quiz (Week 2), the difference was not statistically significant (p = 0.118), suggesting that ChatGPT's impact on retention is comparable to that of other resources [13].

The study's small sample size (N = 33) may have been underpowered to detect subtle differences in retention, and a larger cohort could yield statistical significance. The students' survey results (Appendices) corroborated these findings, showing no significant difference in perceived retention between groups, regardless of the resource used.

There were no significant differences in the time spent completing the quizzes between the groups in both Week 1 and Week 2 (Table 2), indicating that none of the resources, including ChatGPT, provided a measurable advantage in terms of efficiency. However, while no objective time advantage was observed, students in Group A perceived ChatGPT as enhancing their efficiency during the quiz process (Appendices). Although it was hypothesized that ChatGPT's ability to offer quick, personalized explanations would reduce the time needed for quiz completion, the data did not support this [14]. Despite the absence of measurable differences in quiz efficiency, students in Group A perceived ChatGPT as enhancing their quiz efficiency, reported high satisfaction with the tool, and found it helpful for learning, although they expressed mixed confidence in relying on AI as a primary educational resource (Appendices).

These mixed perceptions align with recent studies reporting that while medical students generally value AI tools for their convenience and supportive role, many simultaneously maintain concerns regarding accuracy, reliability, and potential dependence on such technologies [5].

According to the survey results, students in Group B (N = 12) expressed generally positive attitudes toward using external resources, with many considering them more effective for the quiz than internal materials. However, they had mixed opinions about whether these resources enhanced their understanding of medical concepts. In contrast, students in Group C (N = 11) were less satisfied with internal resources, although many still recommended them, possibly due to their familiarity with institutional materials (Appendices).

The mixed feelings about internal resources align with our findings, especially regarding the performance of Group C (N = 11) in comparison to Groups A (N = 10) and B (N = 12). When applying Cognitive Load Theory (CLT), we hypothesize that both ChatGPT and external resources more effectively reduce extraneous load than internal resources, as anticipated. However, the absence of significant differences in retention across the groups may arise from a diminished germane load, as students exert less mental effort when ChatGPT delivers immediate answers. While this interpretation is theoretically supported by Cognitive Load Theory, it remains speculative, as Group A demonstrated the highest average Week 2 score, although the difference was not statistically significant (p = 0.118) (Table 2).

Comparable conclusions have been reported that while AI-based educational tools effectively minimize extraneous cognitive load through rapid and concise explanations, they may simultaneously diminish the deeper, germane cognitive processing essential for long-term learning [9].

ChatGPT shows potential in improving short-term learning, but its impact on retention is still unclear. Future research should explore hybrid educational models that integrate AI tools alongside traditional instruction to determine how best to support both immediate academic performance and sustained knowledge retention. Blended approaches that pair the adaptability and speed of AI with the structure and mentorship of human-guided learning have been proposed as a promising direction for medical education, offering a balanced strategy to optimize student engagement and comprehension [17]. Given the rapid pace of AI development, ongoing evaluation of its role in medical training will be essential to ensure effectiveness, educational quality, and alignment with evidence-based pedagogical practices.

Limitations

This study has several limitations. Although the initial comparison of quiz scores was well powered (99%), the sample size (N = 33) was too small to detect subtler differences in retention; post hoc calculations indicate that 72-132 participants would be required to achieve 80% power for these secondary outcomes [18]. The single‑institution design and exclusive focus on first‑year medical students further limit generalizability. Baseline academic indicators (e.g., college GPA) were not collected, so we could not assess whether ChatGPT's impact varied by prior academic achievement; future studies should include such metrics. Results may also differ for more advanced learners (i.e., third‑ and fourth‑year students), whose greater clinical knowledge and query formulation skills could alter the magnitude and nature of ChatGPT's educational effect. Multicenter studies with larger, more diverse cohorts across multiple training levels are needed to clarify the tool's long‑term effectiveness and broader applicability.

Furthermore, the absence of blinding presents opportunities for performance and expectancy biases. Participants' awareness of their assigned groups may have impacted engagement, especially among those using ChatGPT, where initial exposure to AI tools might have introduced novelty bias. Similarly, researchers were not blinded, which may have resulted in subconscious bias despite following a predefined statistical approach [19]. Our survey data rely on self-reported perceptions and are therefore susceptible to response and social desirability biases. Incorporating objective engagement metrics in subsequent investigations would help validate these findings. Subsequent research should employ blinded assessments, such as independent graders or automated scoring, to mitigate these bias concerns.

Finally, while this study provides insights into short-term learning outcomes, it does not explore the potential long-term effects of AI-assisted tools in medical education. Longitudinal studies tracking performance over extended periods would help determine whether AI-generated explanations foster more profound understanding and knowledge retention beyond the examined timeframe here [16,20].

## Conclusions

These findings offer preliminary insight into the use of ChatGPT in medical education. Participants using ChatGPT showed significantly better short‑term performance than those relying on internal resources (e.g., lectures, slides, and electronic textbooks), although performance was comparable to students using external resources such as Google or PubMed. No clear advantage in knowledge retention was observed across groups. Future research should examine how AI tools can be integrated into curricula to support both immediate performance and long‑term retention. Inter‑institutional studies that include learners at different stages of training are also needed to determine whether these findings generalize beyond first‑year students. Hybrid models that pair the adaptability of AI with the structure of traditional instruction may provide a more comprehensive approach to medical learning. As AI technology evolves, its role in medical education should be reassessed regularly to ensure alignment with evidence‑based teaching strategies and learner needs.

## Appendices

Table 3 presents the survey results of students in Group A (ChatGPT-4.0).

**Table 3:** Group A (N = 10): ChatGPT-4.0 Student Survey Results Students in each intervention group completed a survey assessing their perceptions of their assigned learning resource. The surveys measured key aspects, including students' perceptions of their specific resource in terms of helpfulness, impact on efficiency, quality of information, confidence in its reliability, and overall satisfaction. Data are presented as numbers (%) and Likert scale responses summarized using descriptive statistics. Some questions allowed multiple-choice responses; therefore, percentages per question may not sum to 100%. Likert scale responses range from 1 (lowest) to 5 (highest), with higher values indicating greater perceived helpfulness, efficiency, or quality.

Survey Questions	Level	Group A Overall (%)
How frequently did you use ChatGPT as an aid during your first encounter with the formative quiz?	4 - Frequently	75%
	5 - Very frequently	25%
On a scale of 1 to 5, please rate the overall helpfulness of ChatGPT in assisting your understanding of medical concepts during the first encounter of the formative quiz.	4 - Very helpful	62.50%
	5 - Extremely helpful	37.50%
On a scale of 1 to 5, please rate the impact of ChatGPT on your efficiency in acquiring information.	3 - Moderate impact	25%
	4 - Significant impact	37.50%
	5 - Very significant impact	37.50%
On a scale of 1 to 5, please rate the quality of information acquired through ChatGPT.	3 - Moderate quality	37.50%
	4 - Good quality	50.00%
	5 - Excellent quality	12.50%
Overall, do you believe that using ChatGPT enhanced your efficiency as compared to studying without ChatGPT?	1 - Yes, definitely	37.50%
	2 - Yes, to some extent	62.50%
First time use of ChatGPT in your educational studies?	2 - No	100.00%
On a scale of 1 to 5, please rate your overall satisfaction with using ChatGPT as an educational tool.	4 - Satisfied	75.00%
	5 - Very satisfied	25%
How would you compare the effectiveness of ChatGPT as an educational tool to other traditional resources (e.g., textbooks or lectures)?	1 - More effective	37.50%
	2 - Equally effective	25.00%
	3 - Less effective	37.50%
Did ChatGPT enhance your understanding of medical concepts compared to other traditional resources?	2 - Yes, to some extent	62.50%
	3 - Undecided	12.50%
	4 - No, not really	25%
On a scale of 1 to 5, please rate your confidence in the accuracy and reliability of information provided by ChatGPT	1 - Not confident at all	12.50%
	2 - Slightly confident	25.00%
	3 - Moderately confident	50.00%
	4 - Very confident	12.50%
How comfortable are you with relying on ChatGPT as a primary source of information in your medical education?	1 - Very uncomfortable	20%
	2 - Uncomfortable	20%
	3 - Neutral	20%
	4 - Comfortable	40%
Would you recommend the use of ChatGPT as an educational tool to other medical students?	2 - Yes, with reservations	90.00%
	1 - No	10.00%

Table 4 presents the survey results of students in Group B (external resources).

**Table 4:** Group B (N = 12): External Resources Student Survey Results Data are presented as numbers (%) and Likert scale responses summarized using descriptive statistics. Some questions allowed multiple-choice responses; therefore, percentages per question may not sum to 100%. Likert scale responses range from 1 (lowest) to 5 (highest), with higher values indicating greater perceived helpfulness, efficiency, or quality.

Survey Questions.	Level	Group B Overall (%)
Did you find the access to external resources group (e.g., Google, PubMed, or Sketchy) useful during the formative quiz?	1 - Yes	100%
On a scale of 1 to 5, please rate your overall satisfaction with using external resources as an educational tool for the quiz.	2 - Unsatisfied	9.10%
	4 - Satisfied	45.50%
	5 - Very satisfied	45.50%
How would you rate the effectiveness of resources that were used as an educational tool for the quiz?	1 - More effective	36.40%
	2 - Equally effective	63.60%
Did accessing external resources during the quiz enhance your understanding of medical concepts?	1 - Yes, significantly	18.20%
	2 - Yes, to some extent	36.40%
	3 - Undecided	18.20%
	4 - No, not really	27.30%
On a scale of 1 to 5, please rate your confidence in the accuracy and reliability of information obtained through external resources.	2 - Slightly confident	18.20%
	3 - Moderately confident	18.20%
	4 - Very confident	54.50%
	5 - Extremely confident	9.10%
How comfortable are you with relying on external resources as a primary source of information in your medical education?	1 - Very uncomfortable	9.10%
	2 - Uncomfortable	18.20%
	4 - Comfortable	54.50%
	5 - Very comfortable	18.20%
On a scale of 1 to 5, please rate the quality of information acquired through external resources.	3 - Moderate quality	27.30%
	4 - Good quality	45.50%
	5 - Excellent quality	27.30%
Overall, do you believe that using external resources enhanced your efficiency without compromising the quality of acquired information?	1 - Yes, definitely	36.40%
	2 - Yes, to some extent	54.50%
	3 - Undecided	9.10%
Did you find external resources useful as an educational tool in comparison to your curriculum material (i.e., lecture/PowerPoint)?	a. Yes	90.90%
	b. No	9.10%
On a scale of 1 to 5, please rate your overall satisfaction with using external resources as an educational tool.	3 - Neutral	9.10%
	4 - Satisfied	63.60%
	5 - Very satisfied	27.30%
How would you compare the effectiveness of external resources as an educational tool to other traditional resources (e.g., textbooks and lectures)?	1 - More effective	54.50%
	2 - Equally effective	27.30%
	3 - Less effective	18.20%
Did external resources enhance your understanding of medical concepts compared to curricular materials (i.e., lecture/PowerPoint)?	1 - Yes, significantly	45.50%
	2 - Yes, to some extent	18.20%
	3 - No, not really	36.40%
On a scale of 1 to 5, please rate your confidence in the accuracy and reliability of information provided by external resources.	3 - Moderately confident	36.40%
	4 - Very confident	45.50%
	5 - Extremely confident	18.20%
How comfortable are you with relying on external resources as a primary source of information in your medical education?	1 - Very uncomfortable	9.10%
	2 - Uncomfortable	9.10%
	3 - Neutral	18.20%
	4 - Comfortable	36.40%
	5 - Very comfortable	27.30%
Would you recommend the use of external resources as an educational tool to other medical students?	1 - Yes, definitely	45.50%
	2 - Yes, with reservations	45.50%
	3 - Undecided	9.10%

Table 5 presents the survey results of students in Group C (internal resources).

**Table 5:** Group C (N = 11): Internal Resources Student Survey Results Data are presented as numbers (%) and Likert scale responses summarized using descriptive statistics. Some questions allowed multiple-choice responses; therefore, percentages per question may not sum to 100%. Likert scale responses range from 1 (lowest) to 5 (highest), with higher values indicating greater perceived helpfulness, efficiency, or quality.

Survey Questions	Level	Group C Overall (%)
Did you find the access to internal resources (e.g., lecture materials and medical student note-taking service) useful during the formative quiz?	1 - Yes	72.70%
	2 - No	27.30%
On a scale of 1 to 5, please rate your overall satisfaction with using internal resources as an educational tool for the quiz.	1 - Very unsatisfied	18.20%
	2 - Unsatisfied	27.30%
	3 - Neutral	18.20%
	4 - Satisfied	36.40%
How would you rate the effectiveness of resources that were used as an educational tool for the quiz?	1 - More effective	18.20%
	2 - Equally effective	36.40%
	3 - Less effective	45.50%
Did accessing internal resources during the quiz enhance your understanding of medical concepts?	1 - Yes, significantly	9.10%
	2 - Yes, to some extent	36.40%
	4 - No, not really	54.50%
On a scale of 1 to 5, please rate your confidence in the accuracy and reliability of information obtained through internal resources.	2 - Slightly confident	27.30%
	3 - Moderately confident	45.50%
	4 - Very confident	18.20%
	5 - Extremely confident	9.10%
How comfortable are you with relying on internal resources as a primary source of information in your medical education?	2 - Uncomfortable	18.20%
	3 - Neutral	27.30%
	4 - Comfortable	18.20%
	5 - Very comfortable	36.40%
On a scale of 1 to 5, please rate the quality of information acquired through internal resources.	3 - Moderate quality	63.60%
	4 - Good quality	27.30%
	5 - Excellent quality	9.10%
Overall, do you believe that using internal resources enhanced your efficiency without compromising the quality of acquired information?	1 - Yes, definitely	18.20%
	2 - Yes, to some extent	27.30%
	3 - Undecided	9.10%
	4 - No, not really	36.40%
	5 - No, not at all	9.10%
On a scale of 1 to 5, please rate your overall satisfaction with using internal resources as an educational tool?	2 - Poor quality	18.20%
	3 - Moderate quality	36.40%
	4 - Good quality	27.30%
	5 - Excellent quality	18.20%
How would you compare the effectiveness of internal resources as an educational tool to other resources (e.g., Sketchy, Pathoma, ChatGPT)?	1 - More effective	9.10%
	2 - Equally effective	36.40%
	3 - Less effective	54.50%
How comfortable are you with relying on internal resources as a primary source of information in your medical education?	2 - Slightly confident	18.20%
	3 - Moderately confident	54.50%
	4 - Very confident	9.10%
	5 - Extremely confident	18.20%
How comfortable are you with relying on internal resources as a primary source of information in your medical education?	2 - Uncomfortable	9.10%
	3 - Neutral	45.50%
	4 - Comfortable	27.30%
	5 - Very comfortable	18.20%
Would you recommend the use of internal resources as an educational tool to other medical students?	1 - Yes, definitely	36.40%
	2 - Yes, with reservations	45.50%
	3 - Undecided	9.10%
	4 - No, not really	9.10%
